# Lactic acid induced defense responses in tobacco against *Phytophthora nicotianae*

**DOI:** 10.1038/s41598-024-60037-2

**Published:** 2024-04-23

**Authors:** Fan Yan, Junchi Ma, Manjiang Peng, Congfang Xi, Sheng Chang, Ying Yang, Suohui Tian, Bo Zhou, Tao Liu

**Affiliations:** 1 College of Agriculture and Biotechnology, Yunnan Agricultural University, Kunming, 650201 China; 2 National-Local Joint Engineering Research Center On Germplasm Innovation & Utilization of Chinese Medicinal Materials in Southwest China, Yunnan Agricultural University, Kunming, 650201 Yunnan China; 3 Technology Center of China Tobacco Yunnan Industrial Co., Ltd. Kunming, Yunnan, 650201 China; 4 No. 10 Middle School, Guangnan County, Wenshan Prefecture, Wenshan, 663300 Yunnan China; 5 Tobacco Quality Inspection, Department of Raw Material, Hongyun Honghe Tobacco Group, Kunming, 650051 Yunnan China

**Keywords:** Microbiology, Plant sciences

## Abstract

Induced resistance is considered an eco-friendly disease control strategy, which can enhance plant disease resistance by inducing the plant’s immune system to activate the defense response. In recent years, studies have shown that lactic acid can play a role in plant defense against biological stress; however, whether lactic acid can improve tobacco resistance to *Phytophthora nicotianae,* and its molecular mechanism remains unclear. In our study, the mycelial growth and sporangium production of *P. nicotianae* were inhibited by lactic acid in vitro in a dose-dependent manner. Application of lactic acid could reduce the disease index, and the contents of total phenol, salicylic acid (SA), jasmonic acid (JA), lignin and H_2_O_2_, catalase (CAT) and phenylalanine ammonia–lyase (PAL) activities were significantly increased. To explore this lactic acid-induced protective mechanism for tobacco disease resistance, RNA-Seq analysis was used. Lactic acid enhances tobacco disease resistance by activating Ca^2+^, reactive oxygen species (ROS) signal transduction, regulating antioxidant enzymes, SA, JA, abscisic acid (ABA) and indole-3-acetic acid (IAA) signaling pathways, and up-regulating flavonoid biosynthesis-related genes. This study demonstrated that lactic acid might play a role in inducing resistance to tobacco black shank disease; the mechanism by which lactic acid induces disease resistance includes direct antifungal activity and inducing the host to produce direct and primed defenses. In conclusion, this study provided a theoretical basis for lactic acid-induced resistance and a new perspective for preventing and treating tobacco black shank disease.

## Introduction

Black shank, caused by *P. nicotianae*, is one of the most harmful tobacco diseases worldwide^1,2^. At any stage of tobacco growth, black shank disease can attack all tobacco parts, mainly affecting the base of the stem or roots^3,4^. After the pathogen infection, the stem base will appear irregular black spots, and quickly spread to the upper part of the stem, plant leaves gradually yellow from bottom to top, and in severe cases, black spots will surround the whole stem base, the whole plant of tobacco wilting and necrosis^5,6^. This disease occurs in almost all tobacco-growing areas, devastatingly impacting tobacco and leading to severe annual yield losses worldwide^7^. At present, the prevention and control measures for tobacco black shank, such as crop rotation, fungicide application, biological controls, and are unable to achieve significant effects^8^. Fungicides and biocontrol bacteria are used in most cases nowadays, but the long-term use of fungicides leads to a series of problems, such as pesticide residues and environmental pollution, and there are limitations to biocontrol bacteria which restrict the sustainability of flue-cured tobacco industry^5,9,10^. Therefore, exploring more effective and sustainable tobacco disease control methods is urgent.

In the long process of evolution, plants developed structural and induced defense strategies to boost their disease tolerance^11–13^. The success of plant defenses against invading pathogens depends on early pathogen recognition and initiation of the appropriate signaling processes to activate multilevel allied defense responses^14^. The plant defense mechanism is composed of a double immune system. The first line of defense is plant-triggered immunity (PTI) established through extracellular immune receptors^11,12,15^, whose induced defense generally requires the following steps: (1) pattern recognition receptors (PRRs) recognize pathogen-associated molecular patterns (PAMPs), leading to PTI^11,12,15^; (2) activation of early signaling pathways, including accumulation of calcium by influx, ROS and mitogen-activated protein kinase (MAPK) cascade reactions^16^; (3) hormone-mediated signaling pathways that induce downstream reactions mainly dependent on the cross-communication network between SA, JA or ethylene (ET)-mediated signaling pathways^17,18^. Molecules that trigger PTI responses, such as Flg22, induce PTI reaction-related gene expression that causes ROS levels to increase, stomatal closure and callose accumulation^17,19^. However, under increasingly fierce competition between plants and pathogens, pathogens have acquired the ability to encode effectors into host cells, and inhibit PTI by secreting these effectors, thus improving pathogen virulence. These effectors may directly inhibit plant immune-related proteins or change their activity status^20,21^. In response, plants acquire a skill that can also recognize specific effectors of these attackers, called resistance (R) proteins. When cognate R proteins detect pathogen effects directly or indirectly, they trigger a second layer of the immune system: effector-triggered immunity (ETI)^22,23^. ETI usually initiates an hypersensitive response and is conceptually equivalent to classic gene-to-gene resistance^11,12^. Thus, various plant regulatory factors coordinate to form a highly regulated regulatory network. The activation of a plant’s defense system enhances its defense ability against future pathogens and pests, known as induced resistance (IR), which can be achieved through the application of inducers that mimic pathogen invasion^24^. Some chemical inducers used for IR stimulation include organic acid, cyclopeptides and steroid hormone and so on, such as SA, cyclodipeptide, and brassinoids, which have been shown to induce plant disease resistance^25–27^. These inducers limit pathogen development by activating a variety of molecular and host-cell defense responses, including the recognition of signaling molecules such as NO, Ca^2+^, H_2_O_2_ regulation, and the expression of disease-resistant genes, lignin deposition, phenol and callosity accumulation, the hypersensitive response (HR) and complex hormonal signaling (i.e., SA, JA, and ET) networks^14,25,27^. The use of these IR mechanisms to protect plants may be a more environmentally friendly control method because the metabolic investment in plants is reduced compared with constructive defense activation^14,25,27^. There have been many studies on induced plant resistance, which shed light on resistance expression signaling pathways in plant IR action, whose pathways and characteristics are more widely understood^27,28^.

Studies on tobacco resistance to black shank disease through IR are rarely reported. Previous studies have shown that thiamine can significantly up-regulate PR-1 gene expression in tobacco and induce resistance to tobacco mosaic virus (TMV) through the SA signaling pathway^29^. The defensive response of riboflavin to tobacco black shank and bacterial wilt involves the accumulation of two total phenolic compounds, scopolamine and lignin^30^. Lactic acid is a naturally occurring organic acid widely found in human animals and plants. Recent studies have shown that spraying *Chlorella fusca* on leaves can effectively induce resistance in *Arabidopsis* to *Pseudomonas syringae* and displays a good control effect. Moreover, it is also proven that D-lactic acid in *Chlorella fusca* can be used as an inducer to enhance ROS production, corpus callosum deposition and SA and JA signal-related gene expression in *Arabidopsis*^31^. Importantly, the underlying mechanisms by which lactic acid induces plant resistance remain largely unknown, especially *P. nicotianae* infection, which has not been tested previously. In conclusion, this study aims to elucidate the defense response of lactic acid-activated tobacco to induce resistance to tobacco black shank and highlight the innovative application of lactic acid in plant disease control.

In this study, the antifungal activity of lactic acid against *P. nicotianae* was determined, and its potential role in inducing plant disease resistance and related molecular mechanisms were further studied. We tested the H_2_O_2_, SA, and JA contents and defense enzyme activity in tobacco leaves. In addition, we used RNA-Seq analysis of the lactic acid-induced disease resistance molecular mechanism in tobacco. Our results confirm that lactic acid can induce resistance of tobacco to *P. nicotianae* through SA and JA mediated plant defense pathways. This study first elucidates the mechanisms by which lactic acid triggers a defense response and highlights innovative applications of lactic acid in controlling plant diseases.

## Results

### In vitro inhibitory effect of lactic acid on P. nicotianae

**Figure 1:**
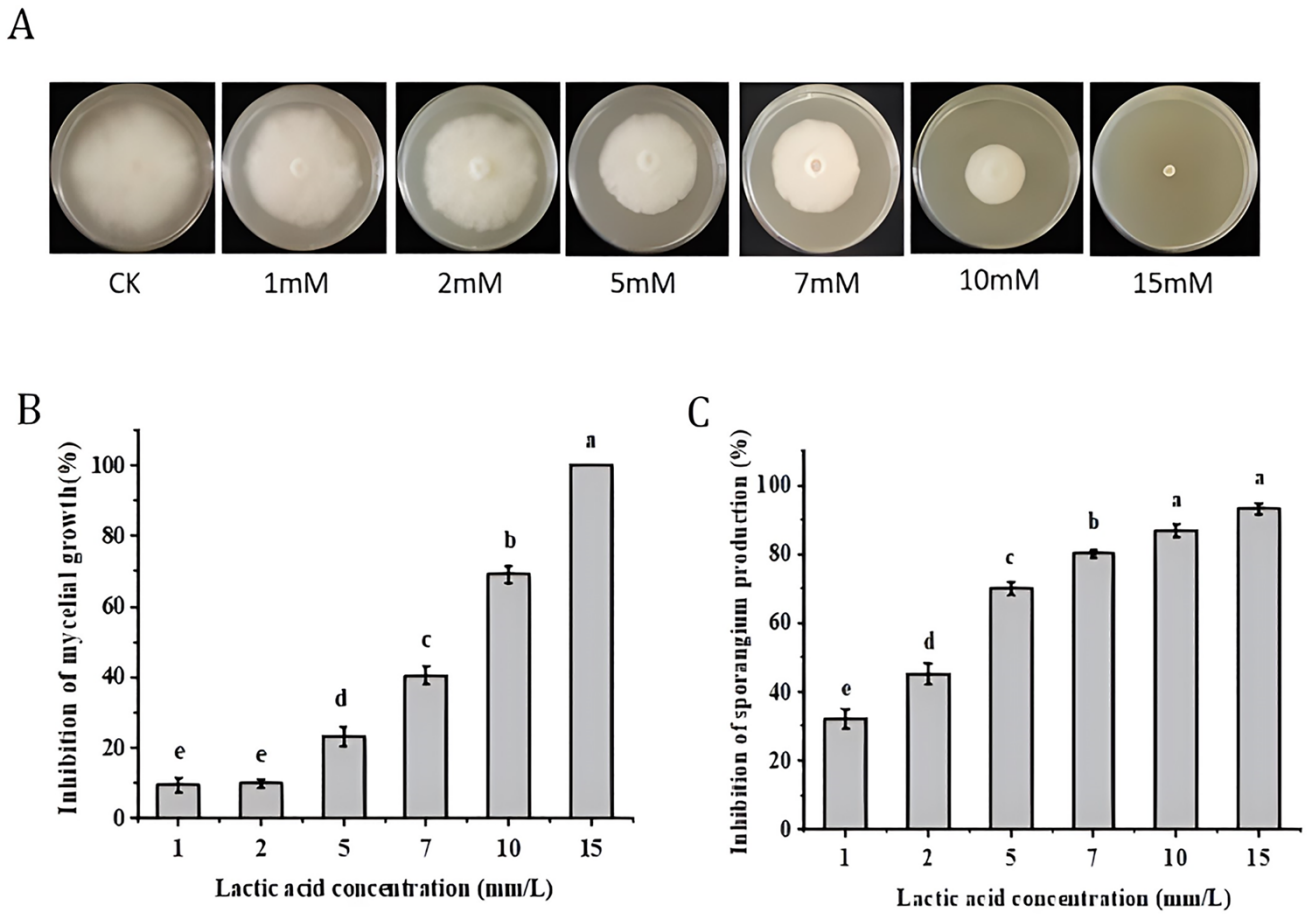
Inhibition of lactic acid on *P. nicotianae *in vitro. (**A**) *P. nicotianae* cultures in Petri dishes illustrating the inhibition of mycelial radial growth with increasing lactic acid concentrations. The mycelial colonies were 15 days old. (**B**) Inhibition of *P. nicotianae* mycelial growth using different lactic acid concentrations. (**C**) Inhibition of *P. nicotianae* sporangium production at different lactic acid concentrations. Data (means ± SE, n = 4) followed by different letters above the bars indicate significant differences (*p* < *0.05*).

In vitro inhibition tests showed that mycelial growth and sporangium production of *P. nicotianae* were inhibited by lactic acid (LA), and inhibition increased with an increase in concentration. LA began to inhibit *P. nicotianae* growth from 1 mM, with an inhibition rate of 69.13% at 10 mM, and complete inhibition of *P. nicotiana* filament growth at 15 mM (Fig. 1A and Fig. 1B). The inhibition rate on sporangium production also showed the same trend with all LA treatments (Fig. 1C). The results showed that LA had an obvious inhibitory effect on *P. nicotianae *in vitro.

### Pot efficacy test of different concentrations of lactic acid on tobacco black shank disease

**Figure 2:**
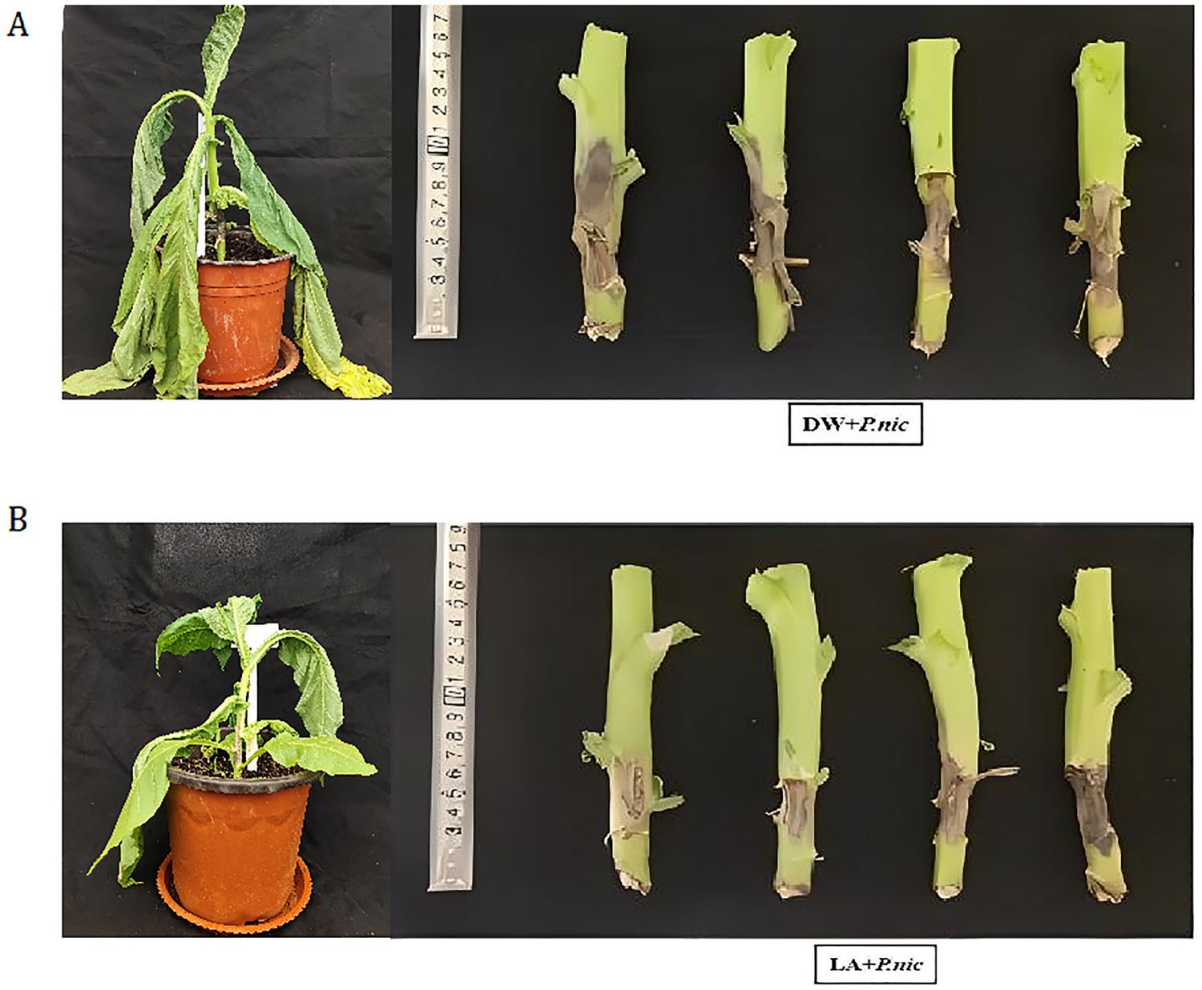
The symptoms of tobacco resistant to *P. nicotianae* treated with exogenous 7 mM lactic acid pretreatment. (**A**) Distilled water was applied as a control, large black necrotic areas in tobacco stems, (**B**) Application of lactic acid reduced the area of disease spots.

All lactic acid concentrations did not significantly affect tobacco maximum leaf width, 10 mM–15 mM lactic acid significantly affect tobacco plant height and maximum leaf length, 15 mM lactic acid significantly affect tobacco stem diameter. Under the 10 mM and 15 mM lactic acid treatment, each index shows a slight downward trend (Table 1). The application of low-concentration lactic acid has an excellent growth-promoting effect on tobacco plants, while high concentrations have a negative impact on tobacco plants. Different lactic acid concentration treatments change the average length of lesions size to varying degrees. The disease index of tobacco plants without lactic acid treatment was 69.8, and the disease index decreased significantly from the 5 mM–15 mM treatment (Table 2). According to the morphological results and control effects of different lactic acid concentrations on tobacco black shank, 7 mM lactic acid was selected to induce resistance in pot experiments. At 2 days post inoculation (dpi), small black lesions were observed on the tobacco stems inoculated with *P. nicotianae*, indicating the successful infection by *P. nicotianae*. At 15dpi, 7 mM lactic acid pretreatment significantly inhibited *P. nicotianae* growth compared with the control and reduced the area of disease spots to varying degrees (Fig. 2). Under lactic acid treatment, the average length of disease spots and disease index decreased, and the relative control effect reached 16.04% (Table 3). These results showed that lactic acid could effectively induce tobacco resistance to *P. nicotianae*.

**Table 1:** Effects of different concentrations of lactic acid on growth indexes of tobacco.

Lactic acid concentration (mM)	Plant height (cm)	Stem diameter(cm)	Maximum leaf length (cm)	Maximum leaf width (cm)
0 (CK)	22.60 ± 2.36a	1.63 ± 0.21ab	13.22 ± 2.22ab	5.03 ± 1.75a
1	25.44 ± 1.04a	1.78 ± 0.22ab	18.06 ± 1.06ab	5.66 ± 1.01a
2	24.46 ± 2.50ab	2.10 ± 0.37a	18.83 ± 1.48ab	5.60 ± 2.43a
5	20.80 ± 2.11ab	1.86 ± 0.16a	12.40 ± 1.06ab	3.72 ± 0.93a
7	20.10 ± 0.37ab	1.63 ± 0.36ab	14.36 ± 3.34ab	5.90 ± 1.42a
10	18.70 ± 1.40b	1.76 ± 0.23ab	11.20 ± 2.62b	2.96 ± 1.53a
15	19.77 ± 0.52b	1.12 ± 0.08b	11.67 ± 2.96b	3.50 ± 0.74a

Data (mean ± SD, n = 15) followed by the different letters among treatments indicate significant differences at *p* < *0.05.*

**Table 2:** Effects of different concentrations of lactic acid on tobacco black shank disease.

Lactic acid concentration (mM)	The average length of lesions size (cm)	The disease index	The relative control effec (%)
0 (CK)	7.83 ± 0.71ab	69.80 ± 0.80a	**—**
1	8.20 ± 0.51a	68.60 ± 2.11ab	1.71 ± 0.98c
2	7.66 ± 0.72ab	66.20 ± 1.05ab	5.15 ± 1.52c
5	6.63 ± 0.63ab	64.60 ± 1.60bc	7.44 ± 2.29bc
7	5.76 ± 0.83b	59.80 ± 1.44 cd	14.32 ± 2.06ab
10	6.23 ± 0.27ab	59.80 ± 1.05 cd	14.32 ± 1.51ab
15	6.65 ± 0.49ab	55.20 ± 2.16d	20.91 ± 3.09a

Data (mean ± SD, n = 6) followed by the different letters among treatments indicate significant differences at *p* < *0.05.*

**Table 3:** Effect of 7 mM lactic acid on tobacco black shank.

Treatments	The average length of lesions size (cm)	The disease index	The relative control effect (%)
DW + P.nic	7.86 ± 0.38	68.00 ± 2.30	**—**
LA + P.nic	6.23 ± 0.26	57.60 ± 1.05	16.04 ± 1.51

Data (mean ± SD, n = 15) followed by the different letters among treatments indicate significant differences at *p* < *0.05.*

### Effects of lactic acid priming on H_2_O_2_ content, protein content, total phenolic and lignin content, CAT, POD and PAL activities in tobacco leaves infected with P. nicotianae

**Figure 3:**
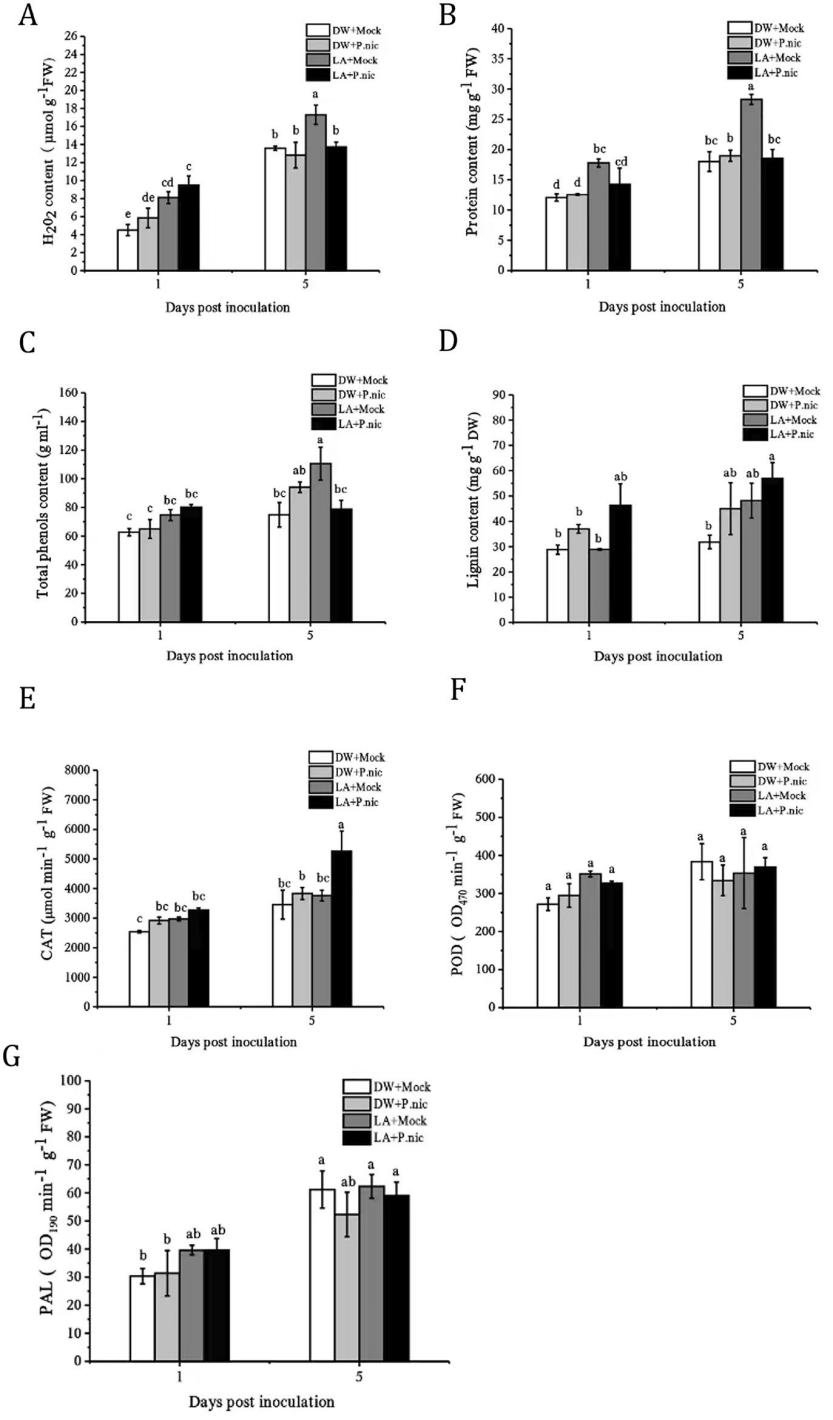
Effect of exogenous 7 mM lactic acid pretreatment for 7 days on (**A**) H_2_O_2_ content, (**B**) protein content, (**C**) total phenolic content, (**D**) lignin content, (**E**) CAT activity, (**F**) POD activity, and (**G**) PAL activity in tobacco leaves after inoculation with *P. nicotianae* (*P. nic*). (DW + Mock) Sprayed with DW/mock inoculation; (DW + *P. nic*) Sprayed with DW/inoculated with *P. nicotianae*; (LA + Mock) Sprayed with lactic acid/mock inoculation; (LA + *P.nic*) Sprayed with lactic acid/inoculated with *P. nicotianae*. Data (means ± SE, n = 4) followed by different letters above the bars indicate significant differences (*p* < *0.05*).

At 1 dpi, compared with the control group, lactic acid/mock inoculation (LA + Mock) treatment and lactic acid/*P. nicotianae* inoculation (LA + *P.nic*) treatment significantly increased H_2_0_2_ content, and lactic acid/mock inoculation (LA + Mock) treatment significantly increased protein content (Fig. 3A-B). Total phenol content, lignin content and CAT activity were no significant difference among treatments at 1 dpi. (Fig. 3C-E). At the 5 dpi, compared with the control, lactic acid/mock inoculation (LA + Mock) treatment significantly increased H_2_0_2_ content by 1.28-fold, protein content by 1.56-fold, and total phenol content by 1.47-fold, lactic acid/*P. nicotianae* inoculation (LA + *P.nic*) treatment significantly increased lignin content by 1.78-fold and CAT activity by 1.52-fold (Fig. 3D and Fig. 3E). There was no significant change in POD activity and PAL activity in all treatments compared with the control (Fig. 3F and Fig. 3G).

### Effects of lactic acid priming on SA, JA and lactic acid contents in tobacco leaves infected with P. nicotianae

**Figure 4:**
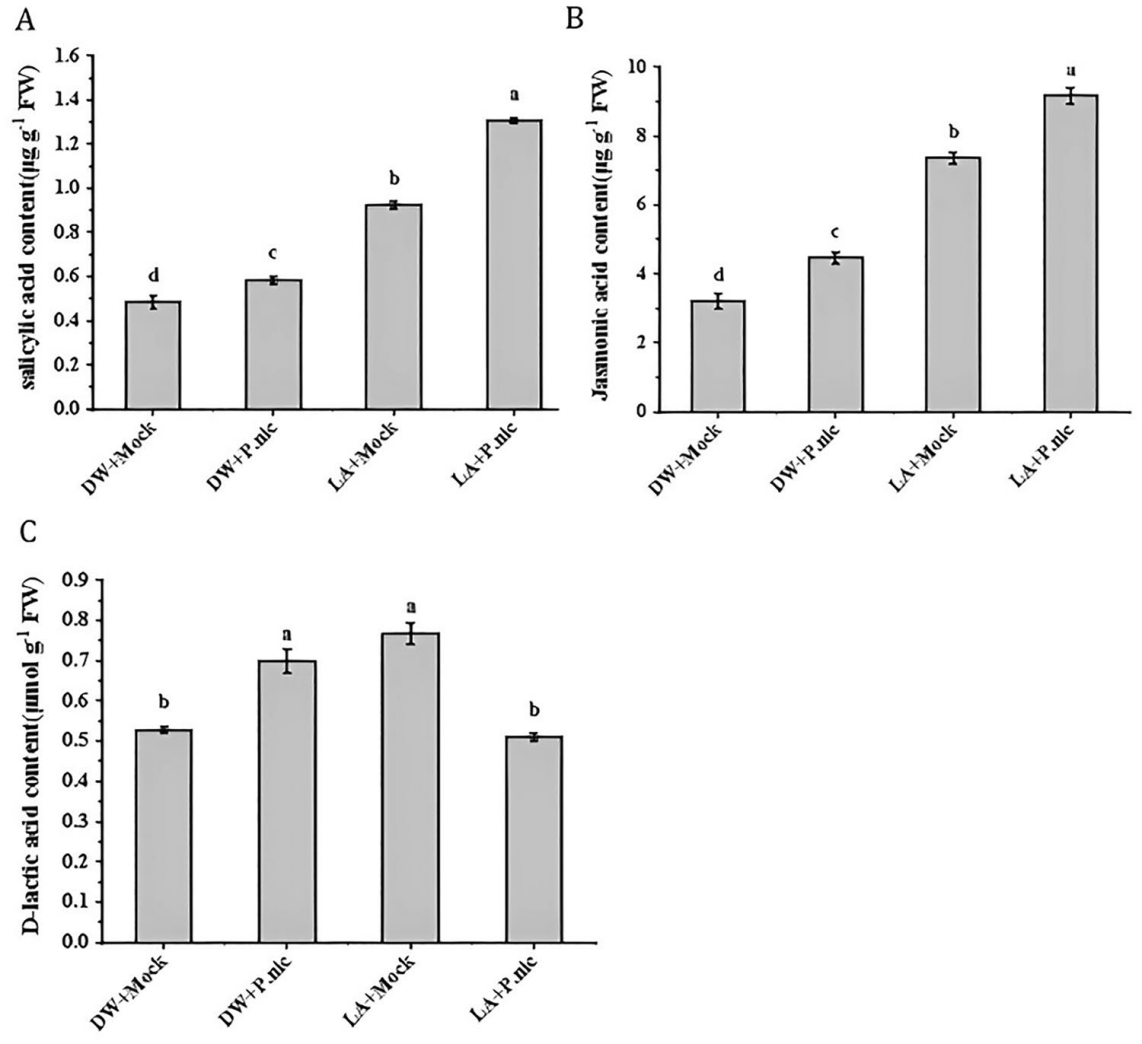
Effect of exogenous 7 mM lactic acid pretreatment on (**A**) salicylic acid content, (**B**) Jasmonic acid content, (**C**) D-lactic acid content in tobacco leaves after inoculation with *P. nicotianae* (*P. nic*). (DW + Mock) Sprayed with DW/mock inoculation; (DW + *P. nic*) Sprayed with DW/ inoculated with *P. nicotianae*; (LA + Mock) Sprayed with lactic acid/mock inoculation; (LA + *P.nic*) Sprayed with lactic acid/inoculated with *P. nicotianae*. Data (means ± SE, n = 4) followed by different letters above the bars indicate significant differences (*p* < *0.05*).

The endogenous SA, JA and d-lactic acid contents in tobacco leaves were determined at 5 dpi. The result is shown in Fig. 4; SA and JA content showed similar content changes among different treatments. When lactic acid was applied alone (LA + Mock) or simultaneously applied with distilled water (DW) and inoculated with *P. nicotianae* (DW + *P.nic*), or simultaneously applied with lactic acid and inoculated with *P. nicotianae* (LA + *P.nic*), the JA content increased by 2.7-fold and 2.86-fold respectively (Fig. 4A and Fig. 4B). In addition, the application of lactic acid alone or inoculation with *P. nicotianae* significantly increased endogenous d-lactic acid content, but endogenous d-lactic acid content did not change significantly under the simultaneous treatment of lactic acid and *P. nicotianae* (Fig. 4C).

### Transcriptomic analysis of tobacco resistance to black shank disease induced by lactic acid

A total of 18 cDNA libraries were tested. Raw reads per sample were 41.9–53.9 million times. After quality filtering, there were still 46,753,124.44 clean reads, ranging from 41.5 million to 52.9 million per sample. According to clean reads quality tests, Q20 for all readings ranged between 96.85% and 97.79%, while Q30 ranged between 91.33% and 93.38%. About 95% of the clean reads were successfully aligned to the *Nicotiana_tabacum* reference genome of tobacco. These results indicated that the gene transcript data were reliable, and suitable for further transcriptomic analysis (Table S1).

### Screening of differentially expressed genes

**Figure 5:**
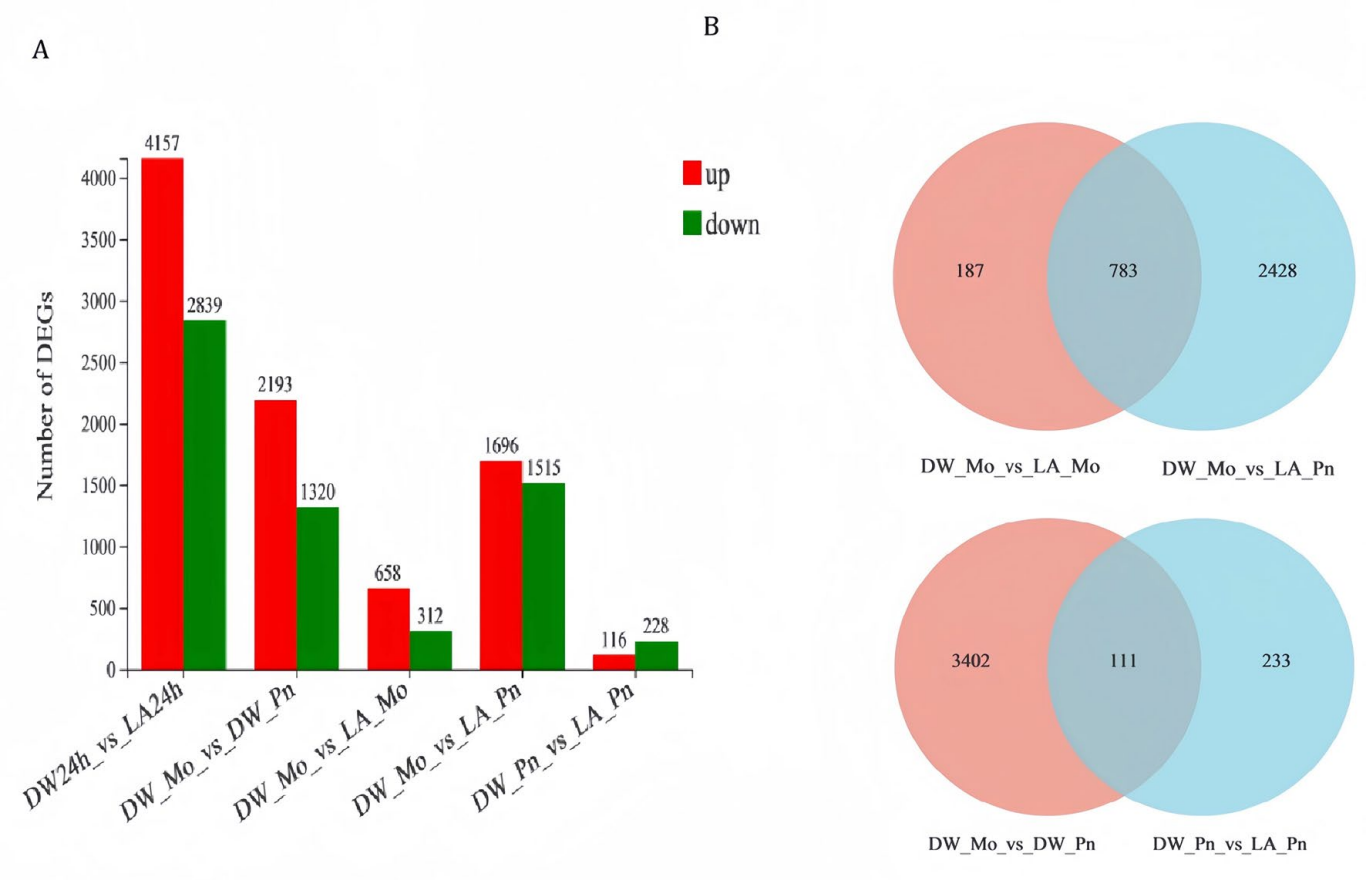
Differential gene expression (DEGs) profiles of tobacco under different treatments. (**A**)Number of DEGs in pairs under different treatments. (**B**) Venn diagram of DEGs. (DW_Mo): after sprayed with distilled water, mock inoculation for 24 h. (LA_Mo): after sprayed with 7 mM lactic acid, mock inoculation for 24 h. (DW_Pn): after sprayed with distilled water inoculated with *P. nicotianae* for 24 h. (LA_Pn): after sprayed with 7 mM lactic acid, inoculated with *P. nicotianae* for 24 h.

The relationship between the 9949 obtained differentially expressed genes (DEGs) was analyzed by clustering. The results showed that the expressions of most DEGs in the treatment group significantly differed from those in the control group (DW24h, DW_Mo) (figure. S2). Compared with DW24h (DW24h vs LA24h), the number of LA24h-DEGs in tobacco leaves was 6996; 4157 genes were up-regulated, and 2839 genes were down-regulated. Compared with DW_Mo (DW_Mo vs LA_Mo), LA_Mo had 970 DEGs, including 658 up-regulated genes and 312 down-regulated genes. Compared with DW_Pn (DW_Pn vs LA_Pn), the number of LA_Pn-DEGs was 344, including 116 up-regulated genes and 228 down-regulated genes (Fig. 5A).

Next, DEGs Venn diagrams of DW_Mo_vs_LA_Mo and DW_Mo_vs_LA_Pn were made, representing 187 DEGs, 2428 DEGs of mock inoculation and inoculation of *P. nicotianae* after spraying lactic acid. DEGs Venn diagram of DW_Pn_vs_LA_Pn and DW_Mo_vs_DW_Pn showed 233 DEGs after the infection with *P. nicotianae* by spraying lactic acid, 3402 DEGs after infection with *P. nicotianae* by spraying DW, and 111 DEGs in total in the two treatment conditions (Fig. 5B).

### GO annotation and KEGG enrichment analysis of DEGs induced by lactic acid in tobacco leaves

**Figure 6:**
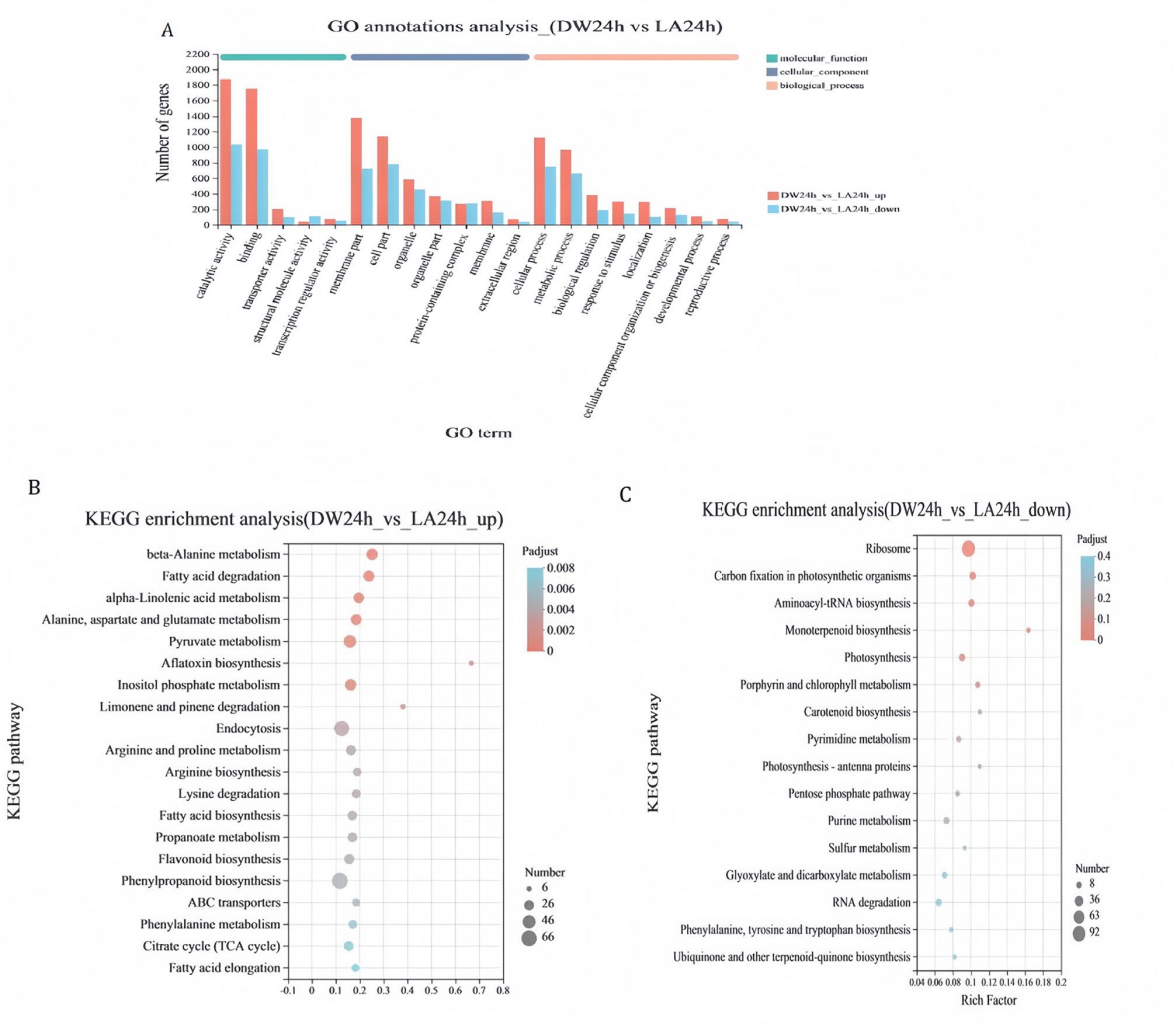
RNA-seq data analysis of DEGs induced by 7 mM lactic acid in Tobacco leaves. (**A**) GO functional annotation analysis, (**B**, **C**) KEGG functional enrichment analysis of up and down-regulated DW24h vs LA24h genes.

A total of 4157 up-regulated genes and 2839 down-regulated DEGs related to DW24h vs LA24h were compared using the gene annotation (GO) database (Fig. 6A). In biological processes, DEGs were mainly involved in metabolic processes, cellular processes and biological regulation. In cell components, DEGs were mainly involved in cell membrane organelles. Among molecular functions, DEGs were mainly involved in catalytic activity and binding.

Further analysis of DEGs in DW24h vs LA24h by Kyoto Encyclopedia of Genes and Genomes (KEGG) showed that up-regulated DEGs were significantly enriched (Q ≤ 0.05) in four pathways were significantly enriched (Q ≤ 0.05, as follows: beta-alanine metabolism, fatty acid degradation, alpha-linolenic acid metabolism, and aflatoxin biosynthesis (Fig. 6B). Down-regulated DEGs were significantly rich in four pathways (Q ≤ 0.1), as follows: ribosome synthesis pathway, carbon fixation in photosynthetic organisms, aminoacyl tRNA biosynthesis, and monoterpenoid biosynthesis. (Fig. 6C).

### GO annotation and KEGG enrichment analysis of DEGs in tobacco resistance to P. nicotianae infection induced by lactic acid

**Figure 7:**
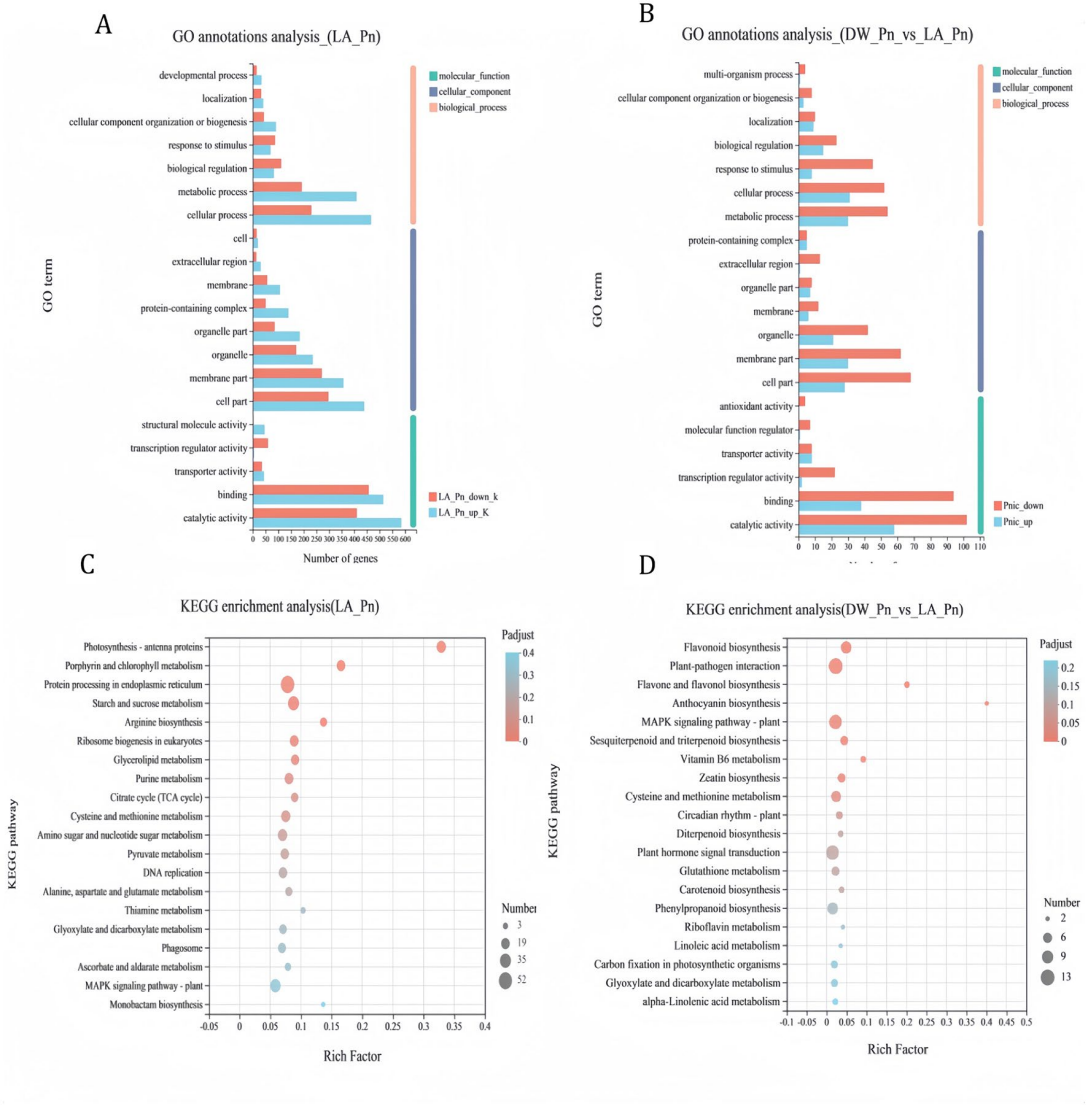
RNA-seq data analysis of DEGs in tobacco resistant to *P. nicotianae* infestans induced by 7 mM lactic acid for 24 h. (**A**) GO function annotation analysis of 2428 DEGs, (**B**) GO function annotation analysis of 344 DEGs, (**C**)KEGG functional enrichment analysis of 2428 DEGs. (**D**)KEGG functional enrichment analysis of 344 DEGs.

We use 2428 DEGs shown in the Venn diagram of DEGs of DW_Mo_vs_LA_Mo and DW_Mo_vs_LA_Pn, the 344 DEGs in the DEGs Venn diagram of DW_Pn_vs_LA_Pn and DW_Mo_vs_DW_Pn were compared using the GO database (Fig. 5B). It was found that these DEGs were mainly involved in biological regulation, cellular processes, and metabolic processes in biological process (Fig. 7A and Fig. 7B).

Next, 2428 DEGs were annotated using the KEGG database to enrich pathways (Fig. 7C). The results showed that these DEGs were enriched in 116 pathways. Photosynthetic-antennae protein enrichment was the highest.

A total of 344 DEGs were annotated using the KEGG database to enrich pathways. The results showed that these DEGs were significantly enriched in five pathway (Q ≤ 0.05) (Fig. 7D), as follows: flavonoid biosynthesis, plant–pathogen interactions, flavone and flavanol biosynthesis, anthocyanin biosynthesis and MAPK signaling pathway-plant. Among them, plant– pathogen interactions and MAPK signaling pathway–plant had the most enrichment genes, and the anthocyanin biosynthesis pathway had the highest rich factor value, indicating that this pathway had the highest enrichment degree. It is also enriched in plant hormone signal transduction and sesquiterpenoid and triterpenoid biosynthesis. Then 223, 111 DEGs in the DEGs Venn diagram of DW_Pn_vs_LA_Pn and DW_Mo_vs_DW_Pn were respectively analyzed for KEGG enrichment (Figures. S3 A and B). It was found that lactic acid-induced tobacco resistance to black shank disease was induced by flavonoid biosynthesis and anthocyanin biosynthesis, and MAPK signaling pathway–plant, plant–pathogen interactions and plant hormone signal transduction were common pathways for plants and lactic acid induced tobacco resistance to black shank disease.

### RNA-Seq revealed the role of four biosynthetic pathways in lactic acid resistance to P. nicotianae

Through KEGG enrichment analysis of the above DEGs, we found that lactic acid- induced tobacco resistance to *P. nicotianae* mainly involved the MAPK signaling and flavonoid biosynthesis pathways through the plant–pathogen interaction plant hormone signal transduction pathway.

**Figure 8:**
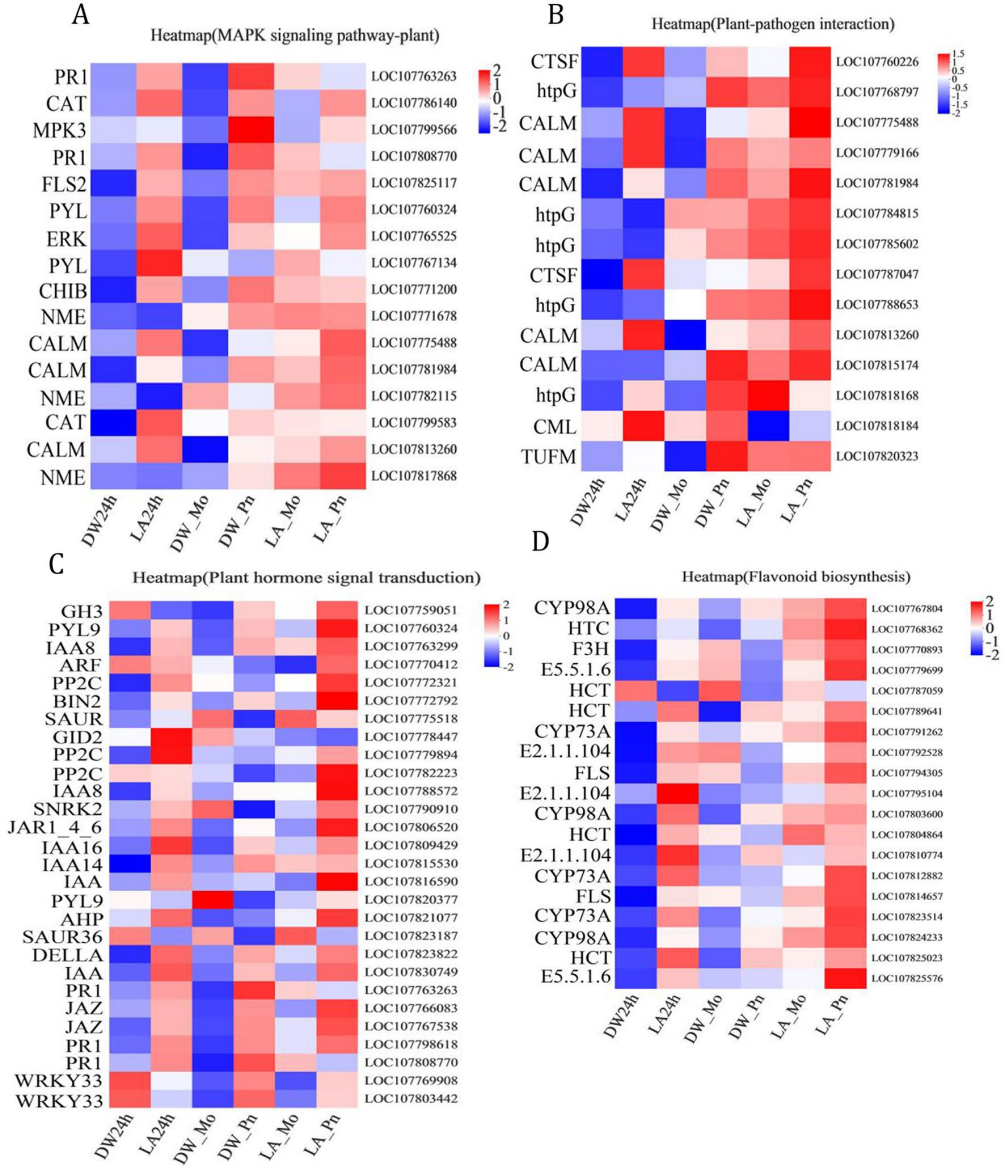
7 mM Lactic acid-mediated (**A**) MAPK signaling pathway, (**B**) Plant-pathogen interaction, (**C**) Plant hormone signal transduction pathway and (**D**) flavonoid biosynthesis pathway of gene heat map analysis.

Signal perception of pathogens and activation of downstream defense signaling molecules, including receptor kinase (RKs), MAPK and modulin-dependent calcium sensor proteins, are critical for plant defense^32^. We conducted a heat map analysis of 16 DEGs with high MAPK signaling pathway expression levels and 14 DEGs related to plant–pathogen interactions (Fig. 8A and Fig. 8B). It was found that the DEGs related to these two pathways were significantly up regulated in the LA24h, DW_Pn, LA_Mo and LA_Pn four treatments. In DW24h vs LA24h, DW_Mo vs LA_Mo and DW_Mo vs LA_Pn, the resistance gene (*PR1*), mitogen-activated protein kinase (*MPK3*), threonine protein kinase (*FLS2*), abscisic acid receptor (*PYL*), mitogen-activated protein kinase (*ERK*) and catalase isoenzyme (*CAT*) gene were significantly up-regulated. In DW_Po vs LA_Pn, the *ERK* gene was significantly up-regulated, *PYL*, *CAT* and *FLS2* remained unchanged, At the same time, *PR1* and *MPK3* were slightly down regulated, suggesting that lactic acid triggers the plant immune response and is involved in the MAPK signaling pathway. In DW24h vs LA24h, DW_Mo vs LA_Mo and DW_Mo vs LA_Pn, cysteine protease (*CTSF*) and calmodulin (*CALM*, *CML*) genes were significantly upregulated, and in DW_Po vs LA_Pn, *CTSF* and *CALM* genes were significantly up-regulated, while *CML* was slightly down-regulated. This result suggests that lactic acid may activate calcium signal transduction, which is further enhanced when plants interact with pathogens.

Heat map analysis was conducted for 28 DEGs involved in hormone signaling pathways (Fig. 8C). In DW24h vs LA24h, transcription factor (*WRKY 33*), PYL, auxin (*IAA*), auxin response factor (*ARF*), protein phosphatase 2 (*CPP2C*), *PR1*, and JA receptor (*JAZ*) gene expressions were significantly up- regulated. In DW_Po vs LA_Pn, *PR1* and *JAZ* were down-regulated, *PYL*, *IAA*, *ARF*, *CPP2C* jasmonate-amide synthase (*GH3*, *JAR1_4_6*, *DELLA*) and serine/threonine protein kinase (*SNRK2*) were significantly up-regulated in *P. nicotianae* infected tobacco. Therefore, this result suggests that lactic acid can activate SA and JA signaling.

Heat map of 19 DEGs related to flavonoid biosynthesis pathway (Fig. 8D): These DEGs were mainly significantly up regulated in LA24h, LA_Mo and LA_Pn treatments. In DW24h vs LA24h, the hydroxy palmitate transferase (*HCT*), methyltransferase (*E2.1.1.104*), cytochrome P450 (*CYP98A*), and trans-cinnamate 4-monooxygenase (*CYP73A*) genes were significantly up-regulated. In DW24h vs LA24h, in addition to the up-regulated genes mentioned above, the naringin, 2-ketoglutarate 3-dioxygenase (*F3H)*, chaleone dihydroflavanone isomerase (*E5.5.1.6*), and flavonol synthase (*FLS*) was significantly up-regulated. These results suggested that lactic acid might synthesize some flavonoid-related substances through the flavonoid pathway to resist tobacco black shank disease.

## Discussion

In vitro inhibition experiments showed that lactic acid could directly inhibit *P. nicotianae* mycelial growth and sporangium production in a dose-dependent manner (Fig. 1), and *P. nicotianae* mycelial growth was completely inhibited by 15 mM lactic acid. Our results were the first to report the direct inhibition effect of lactic acid on *P. nicotianae*, at least indicating that our isolated *P. nicotianae* was sensitive to lactic acid. The control effect of lactic acid was 1.71% ~ 20.91% (Table 1). Pot experiment showed that lactic acid had obvious promoting effect on plant height, stem diameter, maximum leaf length and other agronomic indexes of tobacco at low dose (Table 2). We found that 7 mM lactic acid pretreatment can effectively improve the resistance of tobacco to *P. nicotianae* without toxic effects (Fig. 2, Table 2 and Table 3), indicating that lactic acid could meets the requirements of a plant-induced resistance inducer, and could be further developed and utilized to evaluate its effectiveness in field conditions.

When pathogens infect plants, they lead to the rapid accumulation of ROS (a phenomenon called oxidation explosion), and participate in several important processes related to immune defense and infection^33^. These ROS can directly destroy invading pathogens and participate in coordinating the hypersensitive response (HR). H_2_O_2_ and O_2_^-^ are two major and stable ROS intermediates that act as diffused selection signals to induce gene expression of proteins that are involved in defense and antioxidant processes^34,35^. In this study, the accumulation of H_2_O_2_ in tobacco treated with lactic acid significantly increased (Fig. 3A). Lee et al.^31^ reported that d-lactic acid in *Chlorella fusca* can induce resistance in *Arabidopsis* to *Pseudomonas syringae*, presenting as an ROS eruption. These results suggest that lactic acid pretreatment may be involved in the H_2_O_2_-induced resistance of tobacco by directly killing invading pathogens or stimulating subsequent defense responses.

The ROS stimulated by pathogens is highly reactive and therefore, toxic, harming plant cells. However, excessive H_2_O_2_ levels can also be harmful to plants.

Thus, plants use antioxidant enzyme systems to remove excess H_2_O_2_, among which CAT and POD are the main antioxidant systems protecting cells from oxidative damage^36,37^. In our study, the change in CAT activity was consistent with H_2_O_2_ concentration (Fig. 3), which may mean that CAT degrades H_2_O_2_ at relatively high H_2_O_2_ concentrations.

Lignin is formed upon the effective polymerization of lignin monomers through the consumption of peroxidase and H_2_O_2_, and PAL is the rate-limiting enzyme and key enzyme^38,39^. Lignin deposition can improve the degree of tissue lignification and strengthen plant cell walls^40^. In this study, POD activity did not change in inoculated plants pretreated with lactic acid, but PAL and lignin levels were significantly increased (Fig. 3), suggesting that lactic acid can induce cell wall lignification after pathogen attack. It is well known that plant secondary metabolites are involved in plant defense responses against pathogens and herbivores^41^. Our study also showed an increase in total phenolic content in tobacco plants treated with lactic acid (Fig. 3). Previous studies have shown that d-lactic acid can induce resistance to *Pseudomonas syringae* in *Arabidopsis thaliana*, presenting as ROS eruption and corpus callosum deposition, accompanied by the expression of SA and JA signaling related genes. Our results also showed that lactic acid pretreatment induced a series of defense reactions, including H_2_O_2_ accumulation and significantly increased of defense enzyme activity, SA, JA and total phenolic content, activation of signaling pathways, and then induced lignin production, and increased the cell wall physical barrier to resist penetration by *P. nicotianae.*

SA, JA and ET are major hormones that regulate plant immunity under different biological and abiotic stresses. These three main pathways can interact with other hormonal pathways to create resistance to infection by different pathogens^22^. The role of SA and JA in plant immunity may be antagonistic^22^,^42^. However, there is evidence that salicylic acid and jasmonic acid can cooperate with each other and play synergistic roles in plant immunity^2,43^^.^ This study shows that compared with the control, LA + Mock or LA + *P.nic* treatment significantly increased SA and JA in tobacco leaves (Fig. 4). SA is a necessary endogenous signal molecule for plant SAR production. PAL is a key enzyme in the SA synthesis pathway, and its increased activity is one of the indicators of plant resistance. In our study, at the 1 dpi and 5 dpi, pretreatment with lactic acid increased the PAL activity (Fig. 3). Therefore, lactic acid-induced resistance may be related to both SA and JA pathways.

In the defense response of plants, various hormone signaling pathways are interconnected to produce synergistic antagonistic effects, providing a powerful regulatory network for plants^44^. The upstream pathway of the SA signaling pathway is involved in G-proteins, Ca^2+^, MAP kinase cascades, ROS and NO signaling, and the downstream pathway has been known to generate SA conjugates, ROS, MAPK signaling cascade, WRKYs transcription factors, *NPR1* and *PR* genes. *WRKY* is a key gene in the biological stress response and SA, JA signaling^44–46^. The response of the ABA signaling pathway to various abiotic stresses is mediated by the ABA receptor (*PYR*/*PYL*) and type 2C protein phosphatase (*PP2C*)^44–46^. The interaction of the auxin signaling pathway with various defense signaling systems is more complex and is regulated by Aux/IAA inhibitors and auxin response factor (*ARF*)^47^. Lee et al^31^ showed that lactic acid induces the expression of cysteine-rich receptor-like kinase (*CRKs*), *WRKY* transcription factor genes and SA and JA signaling related genes. The synergy of SA and JA signaling in plant immunity has previously been reported in potatoes, rice, and tobacco^48–50^. In our transcriptomic analysis, lactic acid increased the expression levels of *CAT*, *CALM*, *CMLS*, *MPK3*/*6* and *PR1* in SA signaling pathways, transcription factors *WRKY25*/*33*, *GH3*, *JAR*, *JAZs* and *DELLA* in JA signaling pathways which was consistent with the above results (Fig. 8). As mentioned above, lactic acid significantly increased SA and JA content (Fig. 4). This suggests that lactic acid initiates defense responses in SA and JA signaling pathways involving Ca^2+^ and ROS signaling. In addition, lactic acid up-regulates the expression of *PYL*, *SRK2* and *PP2C* genes in the ABA signaling pathway, *IAA*, *ARF* and *GH3* genes in the auxin signaling pathway. This is consistent with the results reported by Bian et al.^28^ that VMA against pathogens by activating Ca^2+^, ROS, SA, JA, ET, ABA and IAA in the regulatory network signaling pathway.

Our results showed that lactic acid-induced tobacco resistance to black shank disease was also related to flavonoid biosynthesis and anthocyanin biosynthesis. It has been reported that flavonoids play key roles in plant development and defense signaling pathways. It has been reported that flavonoids can bind to protein kinases of pathogens, play a key role in defense signaling pathways, and play an important role in plant growth and development^28^. Du et al. found that exogenous MeJA could significantly up-regulate genes related to flavonoid synthesis and increase the production of flavonoid compounds to reduce fungal infection^51^. The results of this study were consistent with Du et al. The up-regulation of *F3H*, *E5.5.1.6* and *FLS* related to flavonoid biosynthesis under lactic acid treatment (LA24h, LA_Mo) was significantly higher than that in the control group, and the up-regulation degree was higher after pathogen attack (Fig. 8). These results indicated that lactic acid could induce the expression of genes related to flavonoid synthesis and further stimulate the synthesis of some flavonoid related substances to resist *P. nicotianae* during pathogen infection.

Induced resistance includes two mechanisms: direct induction of the defense response and priming of the defense response^52,53^. Direct defense response is locally or systematically induced by inducers stimuli before plants were subjected to any stress. The priming defense response is a process in which plants can be activated only after being stimulated by inducers and attacked by pathogens^54–56^. Previous studies have shown that inducers can activate direct defense responses at high concentrations and trigger the priming defense response mechanisms at low concentrations^54–56^. Boubakri et al. showed that thiamine effectively protects grapevines against *Plasmopara viticola* through a dual action mode of direct antifungal activity and inducing the host to produce direct and primed defense mechanisms^57^. Our conclusion was consistent with the study of Boubakri et al., lactic acid could directly inhibit *P. nicotianae,* and induce the accumulation of a large amount of H_2_O_2_ and the expression of related defense genes in tobacco regardless of pathogen infection. These defense genes were up-regulated or down-regulated in different degrees during subsequent infection by *P. nicotianae.* These results indicated that lactic acid acted as an activator, inducing tobacco to produce a direct defense response, and priming the defense response against *P. nicotianae* through activating related signaling pathways.

## Conclusion

**Figure 9:**
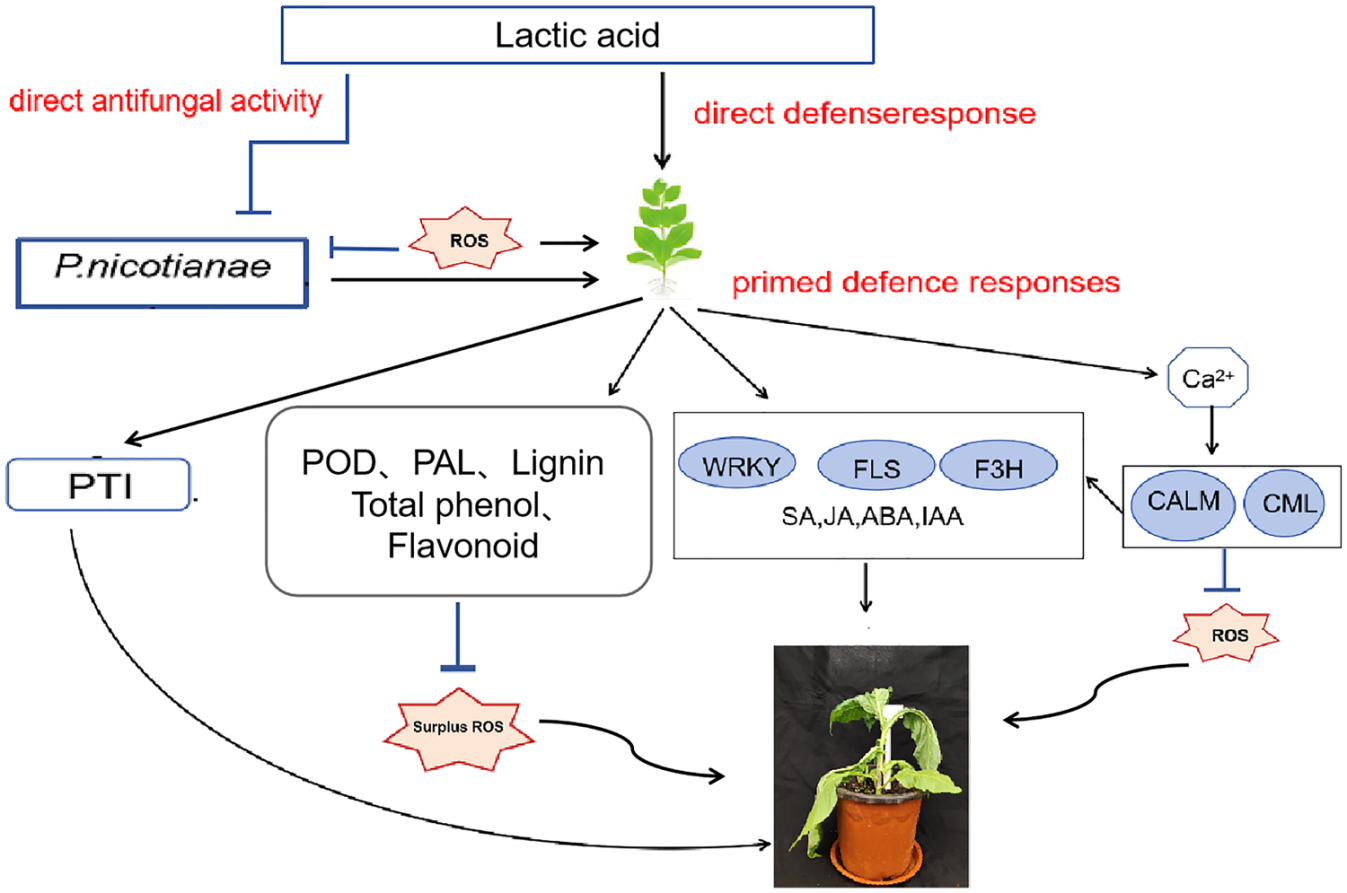
Schematic diagram of molecular mechanism of lactic acid induced tobacco resistance to* P. nicotianae*.

In this study, combining our results, a complex regulatory network was used to describe the molecular role of lactic acid in enhancing tobacco resistance to black shank disease (Fig. 9). We found that when *P. nicotianae* attacked tobacco plants, they could trigger PAMP-triggered immunity (PTI) through the plant hormone signaling pathway, MAPK and plant–pathogen interactions. Lactic acid stimulates tobacco to produce a direct defense response and primes the defense response after subsequent pathogen attack. During the primed defense responses, lactic acid stimulates an increase in intracellular Ca^2+^ and activates a series of calcium-binding proteins that inhibits ROS-generating enzymes. At the same time, stimulus-specific downstream signal transduction was activated. Lactic acid induces the synthesis of some hormones (SA, JA, ABA, TAA) and the expression of genes related to signal transduction, and coordinates the expression of other hormones in the defense network; lactic acid induces the expression of transcription factors (*WRKY*) and related to flavonoid synthesis genes (*FLS*, *F3H*) to resist disease stress. To remove ROS, lactic acid can also induce changes in the activity of some antioxidant enzymes (POD, PAL), as well as the accumulation of representative non-enzymatic antioxidants, such as phenolic compounds, flavonoids and lignin.

In conclusion, the results of this study indicate that lactic acid can probably induce resistance to black shank disease in tobacco plants under greenhouse control through a dual mode of action of direct antifungal activity and induction of host defense mechanisms. Lactic acid can improve tobacco disease resistance by regulating the antioxidant system, plant hormones, transcription factors, flavonoid metabolism, signal molecules and other mechanisms. In this study, lactic acid was found to play an essential role in improving tobacco disease resistance, which laid a theoretical foundation for effective prevention and treatment of tobacco black shank disease.

## Materials and Methods

### Plant material and inoculum preparation

These experiments were conducted at Yunnan Agricultural University from May to August 2021. The experimental material was tobacco; thecultivar was Honghua Dajinyuan (HD). The seeds (donated by the Yunnan China Tobacco Industry Co., Ltd.) were sown in seedling trays, and the seedlings were transferred to pots 30 cm in diameter, after 6 weeks of growing and culturing in a greenhouse (25 ± 3 ℃). All experiments were administered within three weeks after seedling transplantation.

In previous experiments, we isolated and maintained the *P. nicotianae* strain, and the *P. nicotianae* strain was sequenced using a polymerase chain reaction and compared with sequences in the National Center for Biotechnology Information databases^58^. The strain was routinely recultured on potato dextrose agar (PDA: 200 g of potato, 20 g of glucose and 15 g of agar in 1000 mL water) at 27 ℃ in the dark for 15 days^59^.

### Inhibition of different concentrations of lactic acid on P. nicotianae in vitro

The effect of lactic acid on *P. nicotianae* mycelial growth was evaluated on PDA plates according to a method reported previously^60,61^. The lactic acid solution was filtered through a 0.45 μm microfiltration membrane, and PDA containing different concentrations of lactic acid (0, 1, 2, 5, 7, 10, and 15 mM) were prepared. To ensure uniform dissolution of high lactic acid concentration in the medium, the sterilized PDA medium was maintained at about 50 ℃ and shaken gently. Next, the hyphal disks (7 mm diameter) of *P. nicotianae* cultured for 15 days were inoculated on PDA plates containing different lactic acid concentrations, and no lactic acid was added to the control culture. After incubation at 27 °C in the dark for 15 days, the colony diameter was measured to calculate the inhibition rates. Inhibition rate (%) = [(the diameter of control colony − treated colony)/the diameter of control colony] × 100%.

According to reported method^62^, the inhibition effect of lactic acid on the sporangia of *P. nicotianae* was determined. A raw lactic acid solution was mixed with 0.1% KNO_3_ to prepare the induced sporulation solution containing different lactic acid concentrations. The concentrations of induced sporulation fluid were 1, 2, 5, 7, 10, and 15 mM, respectively. The hyphal disks of *P. nicotianae* cultured for 15 days were placed in a petri dish with induction solution, and no lactic acid was added to the control culture. These were cultured at 27 °C in the dark for 48 h. The agar medium, with a thickness of about 1mm, was cut from the lower part of the fungal hyphae disc in parallel and placed on a slide. The number of sporangia was observed and recorded under a 10 × 20 light microscope. Three biological replicates were performed for each experiment.

### Pot experiment of different lactic acid concentrations on tobacco black shank disease

Following a method reported previously^63^, pot experiments were used to test the effect of different lactic acid concentrations on tobacco black shank disease. Tobacco plants were pretreated with different concentrations of lactic acid and inoculated with pathogen to determine the effect of lactic acid on tobacco black shank disease and tobacco growth and development. When tobacco plants grew to 5–6 leaves, the leaf surface and stem base wered sprayed lactic acid of different concentrations, and *P. nicotianae* was inoculated using the mycelium block basal–trauma inoculation method^64^. First, the tobacco plant petiole base was scratched with a blade, then the hyphal disks (7 mm diameter) of *P. nicotianae* were placed on the wound surface and moisturized with sterile cotton, inoculating sterile PDA medium as a blank control.

After 10 days, the length of lesion size on the stem was measured, the disease grade was recorded, and the control effect was calculated. It was recorded as 0 d on the first application day, and the increase in each morphological index was statistically analyzed only 18 d after only application.

### Induction of tobacco resistance against P. nicotianae using 7 mM lactic acid

Lactic acid treatment: According to the morphological results and control effects in the pot experiment testing different lactic acid concentrations on tobacco black shank, 7 mM LA was selected for the following experiment. LA was sprayed evenly on tobacco leaves, and distilled water DW was used as a control. LA was sprayed every 2 days, three times in total. Leaves were detached from the same layer of each plant for assays at 24 h after the third treatment. The total induction time was 7 days. Experimental design and inoculation of *P. nicotianae*: 2 days after the third spray of DW or LA on the leaves, *P. nicotianae* was inoculated. The test design was as follows: (a) Control: Sprayed with DW/mock inoculation (DW + Mock); (b) Sprayed with DW/inoculated with *P. nicotianae* (DW + *P.nic*); (c) Sprayed with lactic acid/mock inoculation (LA + Mock); (d) Sprayed with lactic acid/inoculated with *P. nicotianae* (LA + *P.nic*).

At 15 dpi, the disease state was investigated, and the disease grade was recorded in each treatment. The classification standards and investigation methods of diseases were carried out according to GB/T23222-2008^65^; Classification and Investigation Methods of Tobacco Diseases and Pests. Ten tobacco plants were treated using each treatment, and this experiment was replicated three times. Leaves were taken at 1 and 5 dpi, respectively, and immediately frozen in liquid nitrogen, and stored at − 80 °C until used for enzyme analysis and transcriptomic analyses.

### H_2_O_2_ content, protein content, total phenol content, lignin content, lactic acid content, and enzyme activity determination

H_2_O_2_ content, protein content, total phenol content, lignin content, lactic acid content and the activities of CAT, PAL, and POD were determined using an H_2_O_2_ content assay kit (Suzhou Grace Biotechnology Co., Ltd., Suzhou, China), a protein assay kit (Suzhou Grace Biotechnology Co., Ltd., Suzhou, China), a total phenol assay kit (Suzhou Grace Biotechnology Co., Ltd., Suzhou, China), a lignin assay kit (Suzhou Grace Biotechnology Co., Ltd., Suzhou, China), a lactic acid assay kit (Suzhou Grace Biotechnology Co., Ltd., Suzhou, China) and the CAT, PAL and POD activity assay kit (Suzhou Greys Biological Technology Co., Ltd, Suzhou, China), respectively, H_2_O_2_ content, protein content, total phenol content, lignin content, lactic acid content and enzyme activity were determined precisely according to the instructions in these kits.

### SA and JA measurements

The SA and JA contents in the tobacco leaves of seedlings were measured using high-performance liquid chromatography (HPLC). SA content was determined as described in previous studies with some slight modifications^66,67^. JA content was determined based on the method of Liu et al.^68^. The same part of tobacco leaves under different treatments was sampled, each treatment was repeated three times, and the sterilized scissors were used for picking. The external standard method is used for the quantitative analysis of SA and JA.

Determination of SA content: weigh 0.1 g of sample, grind, add 1 mL of pre-cooled 70% methanol and soak overnight at 4 °C. Centrifuge 8000 g for 10 min, take the supernatant, and extract the residue with 0.5 mL extraction solution for 2 h. After centrifugation, remove the supernatant, combine the supernatant twice, and then filter through 0.45 µm membrane for machine detection. Liquid chromatography conditions: Shimadzu LC-20AT high performance Liquid chromatograph, C18 reversed phase column (250 mm * 4.6 mm, 5 µm), mobile phase A: methanol, B: 0.1% acetic acid water, A: B 35:65, injection volume 10 μL, flow rate 0.8 mL/min, column temperature 35℃, sample removal time: 40 min, UV detector wavelength was 302 nm. The retention time is 8.33 min.

JA content was measured^69^, 0.1 g sample was accurately weighed, 1 mL extraction solution was added and ground into pulp, 1 mL 2 M HCl was added to adjust pH to 2.5–3.0, and then transferred into EP tube after shock and mixing, ultrasonic extraction for 30 min, centrifugation for 8000 g, supernatant was removed, 0.3 mL extraction solution was added into the residue, and the supernatant was combined twice. Dry the nitrogen blower in ice bath, add 0.3 mL mobile phase to redissolve, filter the membrane, and test on the machine. Liquid chromatography conditions: Wufeng LC-100 high performance liquid chromatograph, C18 reversed-phase column (250 mm * 4.6 mm, 5 µm), mobile phase A: acetonitrile, B: 1% formic acid in water, A:B 45:55, injection volume of 10 μL, flow rate of 1 mL/min, column temperature of 35 ℃, the walk-around time of :40 min, and the wavelength of the ultraviolet detector was 210 nm. The retention time is 24.75 min.

### RNA-Seq and data analysis

According to the reported method^70^. RNA-Seq and data analysis were carried out. The treatments used for transcriptome sequencing were as follows: 7 mM lactic acid treatment for 24 h (LA24h) or DW treatment for 24 h (DW24h), after spraying with 7 mM lactic acid, and inoculated with *P. nicotianae* for 24 h (LA_Pn) or mock inoculation for 24 h (LA_Mo), after spraying with DW, and inoculated with *P. nicotianae* for 24 h (DW_Pn) or mock inoculation for 24 h (DW_Mo). Leaves from the same part of three plants were collected and immediately frozen using liquid nitrogen and stored at − 80 °C for transcriptome sequencing.

Total RNA was extracted using TRIzol reagent (Invitrogen), and each sample was purified with Plant RNA Purification Reagent (Invitrogen) according to the manufacturer’s instructions. The samples were analyzed by 2100 Agilent Technologies for RNA size quantification and quality control. Each transcriptome library consists of 1 μg total RNA. According to the manufacturer’s instructions, the TruSeqTM RNA sample preparation kit (Illumina, Inc., San Diego, CA) was used to generate a sequencing library. The library preparation and high-throughput RNA sequencing were completed using HiSeq 4000 equipment (Illumina) operated by Major Genome Center, Shanghai, China.

RawData were obtained by sequencing on an Illumina high-throughput sequencing platform, and CleanData were obtained by removing linker sequences and low-quality reads. CleanData and tobacco reference genome (https://www.ncbi.nlm.nih.gov/genome/425?Genome_assembly_id=274804) to obtain MappedData. RSEM software was used to analyze the differential expression of transcripts. Then, selected DEGs were annotated using the GO function and enriched using the KEGG pathway. DEGs were screened using DESeq2, and p < 0.01 and a multiple change (FC) ≥ 2 was selected as the threshold for significant differentially expressed genes (SDEGs). Based on the GO and KEGG databases, the functional enrichment analysis of all SDEGs was carried out using GoTools (github.com/Tang Haibao/GoTools) and Kobas software (kobas.cbi.pku.edu.cn/home.do). In Mapman software, absolute log2 times change data are used, and a heat map is constructed using the gene cluster method.

### Statistical analysis

The repetitions and replications for each assay are repeated in triplicate. Data were input into Microsoft Excel 2016 software, and Origin 2018 was used to analyse the data. These graphs were generated using Origin 2018. Origin 2018, with values expressed as means ± SE. Significant differences between means were analyzed at *p* < *0.05* using a one-way analysis of variance (ANOVA).

### Ethics approval and consent to participate

The use of plant parts in the present study complies with international, national and/or institutional guidelines for plants.

### Supplementary Information

Supplementary Figures.

Supplementary Table S1.

## Data Availability

The datasets generated and/or analysed during the current study are available in the NCBI repository, ACCESSION NUMBER TO DATASETS IS SRP382131.
