# Supplementary material for: Lactic acid induced defense responses in tobacco against Phytophthora nicotianae
Source: Sci Rep. 2024 Apr 23;14:9338. doi: 10.1038/s41598-024-60037-2 (PMC11039699; doi:10.1038/s41598-024-60037-2)
Supplement: Supplementary file 1 — Supplementary Figures. [file 41598_2024_60037_MOESM1_ESM.docx]

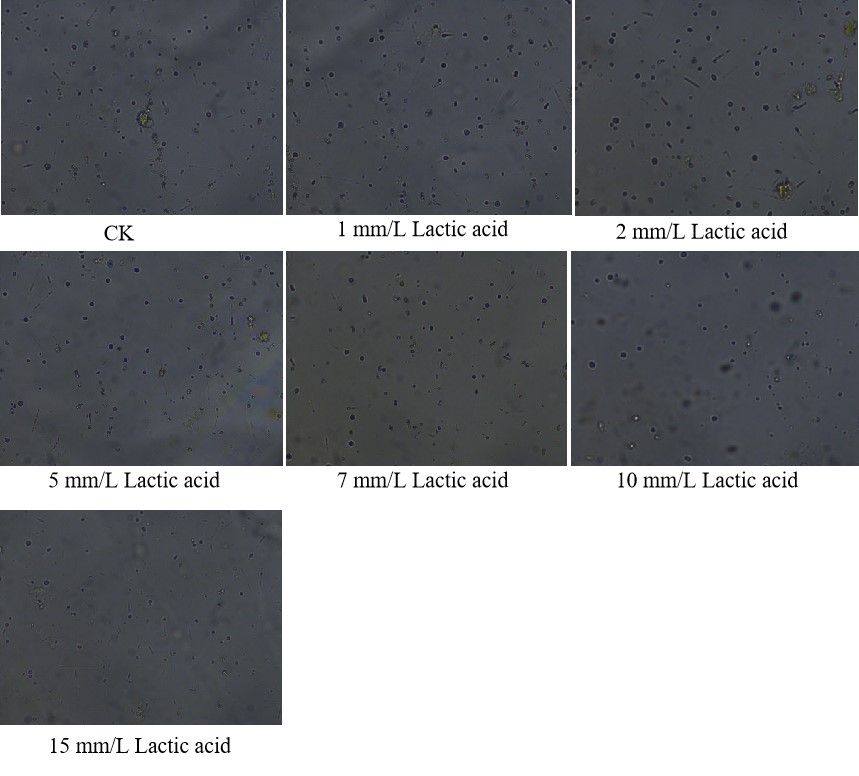


**Figure S1.**Inhibition of sporangium by lactic acid. (40*10)


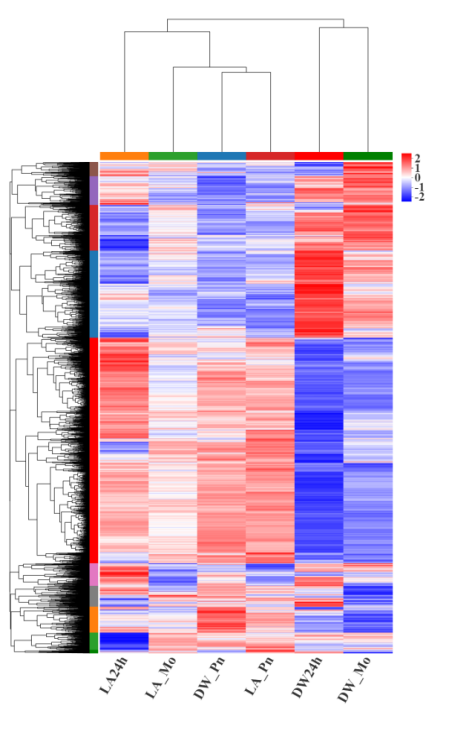


**Figure S2.**Cluster analysis of tobacco differential genes under different treatments


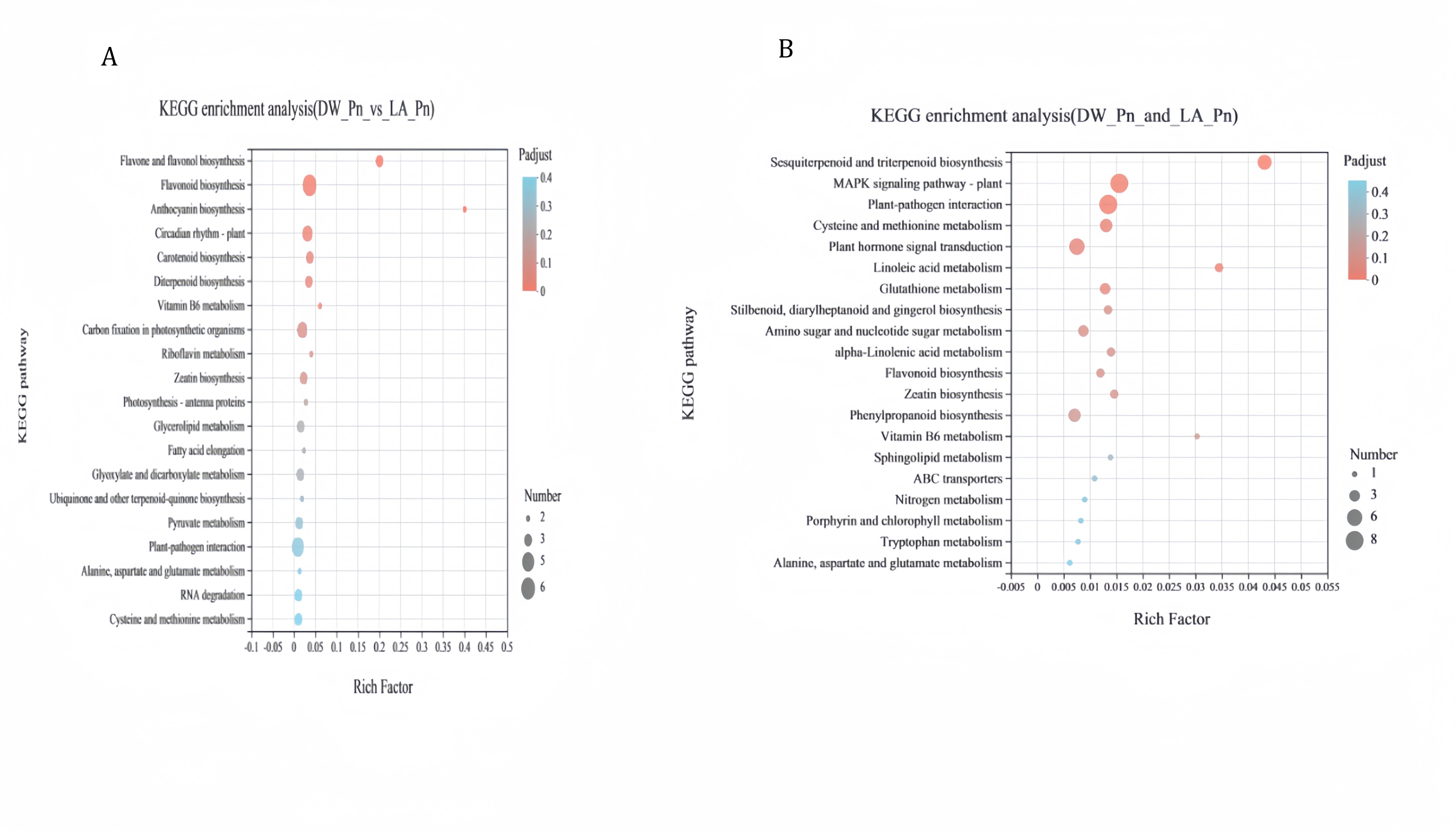
 **Figure S3.** RNA-seq data analysis of DEGs in tobacco resistant to P. nicotianae infestans induced by lactic acid. (A) KEGG functional enrichment analysis of 233 DEGs. (B)KEGG functional enrichment analysis of 11 DEGs.


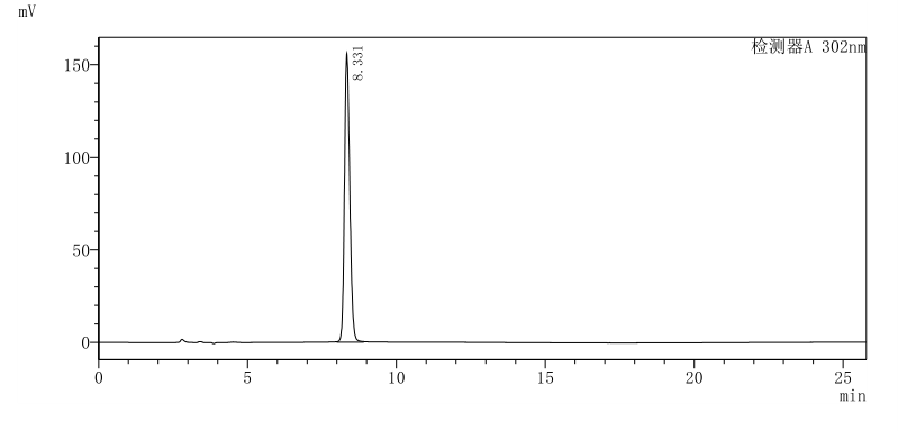


SA standard product spectrum


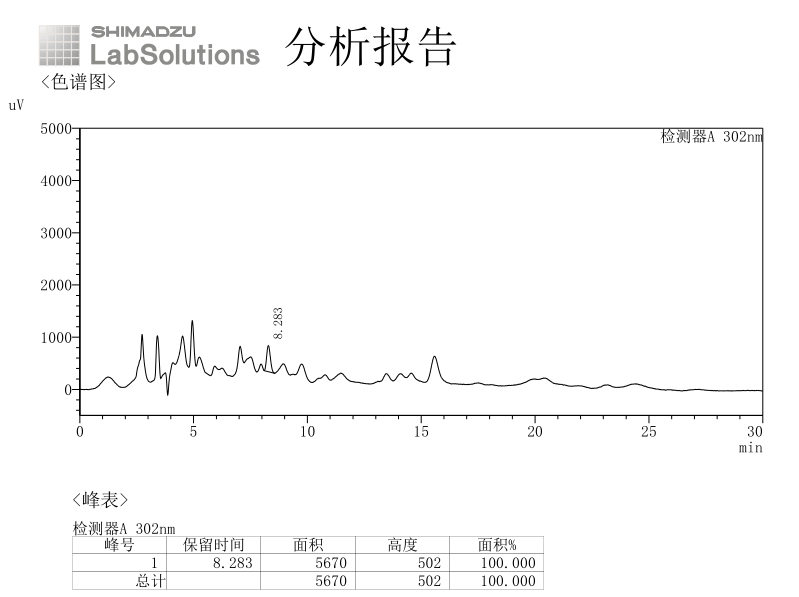


SA sample spectrum


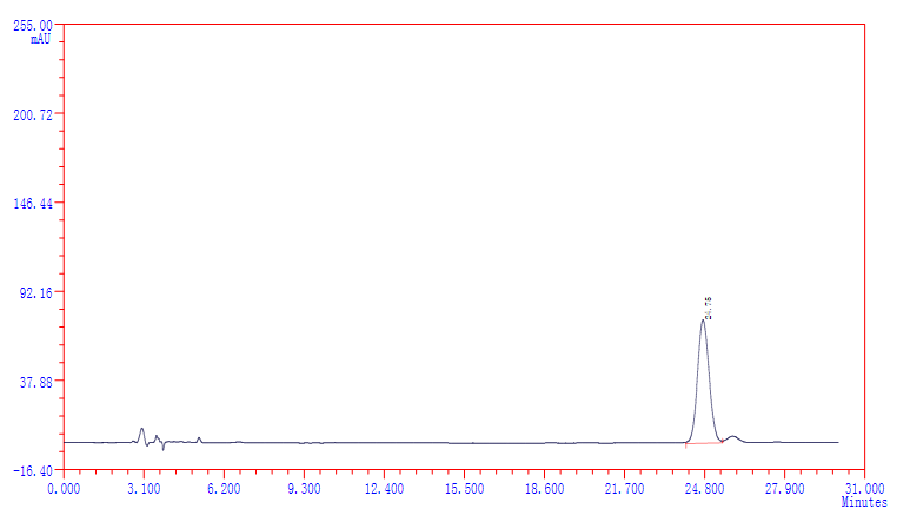


JA standard product spectrum


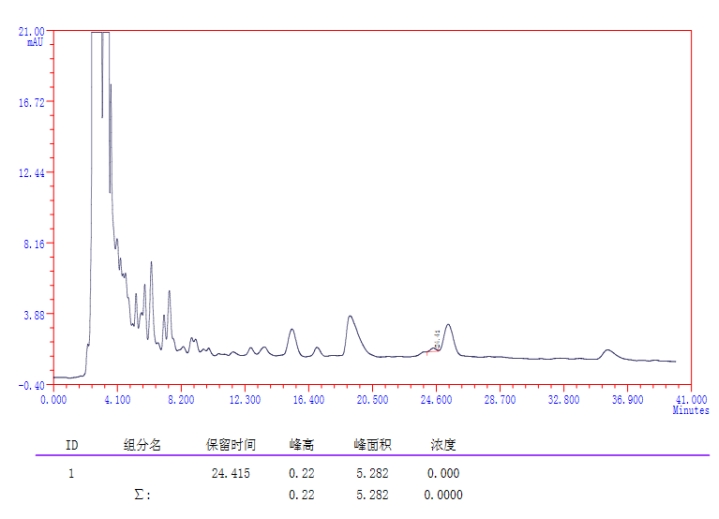


JA sample spectrum

**Figure S4.** SA, JA High Performance Liquid Chromatography
