# Supplementary material for: Lactic acid induced defense responses in tobacco against Phytophthora nicotianae
Source: Sci Rep. 2024 Apr 23;14:9338. doi: 10.1038/s41598-024-60037-2 (PMC11039699; doi:10.1038/s41598-024-60037-2)
Supplement: Supplementary file 2 — Supplementary Table S1. [file 41598_2024_60037_MOESM2_ESM.docx]

**Tab S1** The reads count from eighteen cDNA libraries in RNA-seq.

| Sample | Raw reads | Clean reads | Clean bases | Error rate(%) | Q20(%) | Q30(%) | GC content(%) |
| --- | --- | --- | --- | --- | --- | --- | --- |
| LA_Pn1 | 46319294 | 45969686 | 6798532600 | 0.0267 | 97.35 | 92.6 | 43.34 |
| LA_Pn2 | 45659636 | 45272768 | 6628931881 | 0.0271 | 97.19 | 92.26 | 43.83 |
| LA_Pn3 | 42768630 | 42403720 | 6215350663 | 0.0279 | 96.85 | 91.49 | 43.82 |
| LA_Mo1 | 46070622 | 45645190 | 6703882249 | 0.0271 | 97.15 | 92.19 | 43.26 |
| LA_Mo2 | 46281140 | 45835046 | 6747173220 | 0.027 | 97.18 | 92.28 | 43.48 |
| LA_Mo3 | 46711894 | 46328412 | 6811830491 | 0.0267 | 97.33 | 92.64 | 43.38 |
| DW_Pn1 | 41906134 | 41522208 | 6105561123 | 0.0281 | 96.79 | 91.33 | 43.64 |
| DW_Pn2 | 46196226 | 45815110 | 6765419737 | 0.0265 | 97.37 | 92.75 | 43.66 |
| DW_Pn3 | 46183182 | 45777018 | 6717556647 | 0.0267 | 97.3 | 92.55 | 43.6 |
| DW_M1 | 47747480 | 47330142 | 6993968581 | 0.0264 | 97.43 | 92.87 | 43.38 |
| DW_M2 | 49363332 | 48864340 | 7211774471 | 0.0259 | 97.63 | 93.38 | 43.32 |
| DW_M3 | 48962310 | 48446002 | 7124357048 | 0.0261 | 97.53 | 93.14 | 42.99 |
| LA24h1 | 46686714 | 46273994 | 6824470138 | 0.0279 | 96.87 | 91.54 | 43.41 |
| LA24h2 | 48638286 | 48234012 | 7095012714 | 0.027 | 97.19 | 92.28 | 43.61 |
| LA24h3 | 46709720 | 46243726 | 6827260021 | 0.0274 | 97.03 | 91.95 | 43.43 |
| DW24h1 | 49486340 | 48914868 | 7182694788 | 0.0261 | 97.53 | 93.12 | 42.72 |
| DW24h2 | 50614102 | 49771210 | 7433696794 | 0.026 | 97.56 | 93.27 | 42.62 |
| DW24h3 | 53911998 | 52908788 | 7899073967 | 0.0258 | 97.63 | 93.43 | 42.07 |
